# Analysis of Accuracy and Reliability of Different Types of GPS Receivers

**DOI:** 10.3390/s20226498

**Published:** 2020-11-13

**Authors:** Mariusz Rychlicki, Zbigniew Kasprzyk, Adam Rosiński

**Affiliations:** Faculty of Transport, Warsaw University of Technology, Koszykowa 75, 00-662 Warsaw, Poland; mry@wt.pw.edu.pl (M.R.); zka@wt.pw.edu.pl (Z.K.)

**Keywords:** GNSS, GPS quality, positioning error, utility software

## Abstract

There are several known cases of positioning error, leading to serious consequences, sometimes also deadly. Therefore, obtaining accurate position data by means of GPS receivers is paramount. With this perspective, the aim of this study was to test the within-field accuracy of different types of GPS receivers, and to determine their reliability. A proprietary software was used to determine the positioning accuracy of nine different types of satellite receivers. In addition, their reliability was investigated, by including tests aimed at measuring their positioning accuracy in field conditions. Thus, it was possible to determine the probability that these GPS receivers can be in some states (reliability). The developed software solution could be used for further research on a wider group of the same types of satellite receivers. The results of this study could lead to draft a procedure for evaluating and selecting GPS receivers, based on their quality, prior to use. This could have a paramount importance for uses in special purpose vehicles or transport telematics systems.

## 1. Introduction

A GNSS (Global Navigation Satellite System) must be economically viable in order to be used, and, depending on the crop operation, must achieve high values of positioning accuracy. The positioning accuracy of a GNSS is the distance between the position of a point on the Earth’s surface determined by this system and the real one [1]. The positioning accuracy of a GNSS, e.g., GPS, has also become critical for assuring the efficiency and safety of both people and cargo transportation [2,3,4,5]. There are several known cases of positioning error, leading to serious consequences, sometimes deadly ones [6]. Therefore, obtaining precise positioning information by means of GPS receivers is paramount. In this paper, a proprietary software was used to determine the positioning accuracy of different types of GPS receivers. In addition, their reliability was investigated, by including tests aimed at measuring their positioning accuracy in field conditions. The reliability of a GNSS is the probability of performing positioning without failures, in determined conditions and during a specified time interval.

The low reliability of GPS receivers and actions based on high positioning error often lead to hazardous situations [7]. Analyses of various types of damage to devices, as well as operator errors, are paramount for the safety of agricultural work [8]. Key competences in this area include the ability to determine which state of the transportation system, consisting of an operator, a GPS receiver, and a vehicle tracking device, can be perceived as either acceptable or unacceptable from the viewpoint of safety, based on the positioning accuracy. A safer transport system can be built up by increasing its reliability. This can be achieved by improving the reliability of its components or by using redundant structures [9,10]. In the former case, the ultimate goal is to prevent faults, which generates a substantial cost, both in terms of manufacturing and maintenance. In the latter case, redundant infrastructure means better tolerance of failures [11,12], through the extension of the transport system. This consequently generates significant maintenance costs [13,14]. Therefore, GPS quality, which depends on DOP (dilution of precision), the number of visible satellites (having a minimum elevation mask of 10–15°), and positioning accuracy [1,15,16] fed into a transport system including GPS receivers [17,18,19,20], is paramount. In this perspective, an original software solution for analyzing GPS data and determining their positioning accuracy has got a great value.

Positioning accuracy is, among others, the subject of paper [21], whose authors presented results of research about accuracy as a function of HDOP (horizontal DOP, related to longitude and latitude). In that study, NMEA 0183 data were used in order to measure the positioning accuracy of the analyzed GPS receivers. Two identical receivers were used to ensure the reliability of the obtained results. These results showed an almost linear relationship between the positioning accuracy and HDOP. In paper [22] the number of visible GPS and GLONASS (ru. Глобальная навигационная спутниковая система) satellites, as well as DOP, were registered under conditions of uniform distribution of GNSS satellites above the observation point, so that the number of available GNSS signals was limited during the tests. The same test was repeated but under conditions of not uniform distribution of GNSS satellites above the observation point. This research revealed how redundancy, i.e., additional satellites (more than two for determining a 2D position and more than three for sensing a 3D position), positively influence DOP (decreasing) and therefore the positioning accuracy (increasing). As a consequence of using GLONASS, the distribution of satellites above the observation point is more uniform, so that it allows the user to carry out continuous positioning under difficult conditions—something previously impossible when using only GPS. In paper [23] the authors analyzed positions from commercially available GPS receivers and concluded that the standard positioning algorithm used by GPS is inaccurate. The GNSS positioning accuracy could be improved by developing and implementing software, since achieving a better performance by means of satellite receivers using additional satellites is prohibitively expensive. Paper [24] discusses software for acquiring, analyzing, and processing multiple streams of GPS data. This software is capable of distinguishing various data and makes monitoring possible over the internet by using an IP protocol. However this software was missing an analysis of reliability, which would require a tool for determining the probability that a transportation system can assume some states (reliability), something vital from the viewpoint of assuring an efficient and safe transportation of people and cargo. The author of the paper [25] proposed a software for analyzing NMEA 0183 data streams in mobile devices such as smartphones, sending and receiving data via Bluetooth. The end results were the characteristics of the obtained datasets. While the focal point for the author of the above paper was how mobile devices support the NMEA 0183 standard via Bluetooth, at the same time this work failed in explaining GPS positioning errors. In the paper [4], authors focused on a solution for minimizing GPS positioning error, by implementing a generic software. That technique would use the received data for determining reasonably accurate positions. Although this approach could improve the positioning accuracy, it requires at least two GPS receivers whose exact location is known. The authors did not carry out any analysis of reliability of GPS receivers. Whilst the suggested solution does improve the reliability of GPS receivers as a consequence of higher redundancy, it requires the expansion of the current infrastructure at high cost (for both initial investment and subsequent maintenance). In another paper [6], the authors discussed the importance of accurate GPS data for formulating transport policy, based on the example of Belgium and the Brussels capital region. They also proposed some indicators concerning with urban transportation, which can be extracted from a wide collection of GPS trajectories of lorries. Sample data were obtained from GPS receivers mounted on lorries, as these devices had become mandatory in Belgium as part of an automated toll system introduced in 2016.

Another study [26] focused on the techniques aimed at improving the accuracy and reliability of position data, and proposed integrating GPS and BeiDou receivers. The achieved result was a higher number of visible satellites, moving along different orbits, as well as an improved positioning accuracy. That approach also increases the probability of detecting a failure of GNSS [27].

Paper [28] described a solution using four GNSS, i.e.: GPS, GLONASS, BeiDou, and EGNOS. Subsequent research revealed that such a four-constellation positioning system decreases the convergence time (convergence time is the time needed to obtain the maximum possible measurement accuracy for a given technique, counted from the moment the receiver is turned on) by 70% and improves the positioning accuracy by about 25% (compared to only GPS). Hence, that approach is correct and further research and simulations should be carried out.

Yet another technique was proposed in paper [29], i.e., an original system for simultaneously receiving L1 (1575.42 MHz) and L5 (1176.45 MHz) signal frequencies from GPS satellites. That solution is supposed to improve the positioning accuracy and reliability of GPS receivers.

When designing and implementing new solutions for integrating various GNSS, such solutions need to undergo functional and reliability tests, as described in paper [30]. That paper’s authors proposed an approach based on various hypotheses, thus allowing the integration of GPS and GLONASS, in order to increase the positioning accuracy and reliability.

Despite substantial scientific research concerning GNSS, only two approaches were considered. They were the integration of different positioning systems (GPS, GLONASS, BeiDou, and EGNOS) and the development of algorithms that would improve the accuracy and reliability of position data [31,32,33,34]. Therefore, the aim of this paper is to propose an innovative approach, involving the development of an original software, able to obtain position data and analyze the accuracy and reliability [35,36] of nine GPS receivers.

Furthermore, this paper discusses fundamental issues concerning positioning errors, by deeply analyzing GPS data. As the transportation of both people and cargo is highly based on GNSS, this key dependency could lead to the disruption of these transport systems.

## 2. Materials and Methods

In order to determine the positioning accuracy of the analyzed GPS receivers, first the position data needs to be obtained in a format that allows comparison and analysis. An ideal format, yielding the most accurate results, involves the simultaneous acquisition of position data from all the analyzed GPS receivers. This integration is possible, as every GPS receiver communicates with its parent device via RS 232C, USB, or Bluetooth. The position data are transmitted through NMEA 0183 standard, developed by the National Marine Electronics Association (NMEA) as a serial communications protocol between marine navigation devices such as LORAN, OMEGA, TRANSIT, and GPS receivers [37]. It defines an electrical interface, a data transmission protocol and a format of message exchange [37,38]. Every device using NMEA protocol for communication can connect and send data to multiple nodes. The standard serial transmission rate is 4800 b/s, which is more than enough for a correct communication. There are also devices operating at higher data transmission speeds. Messages are sent every second as a row of printable characters ending with a check sum. In the case of GPS receivers used as part of a transportation system, constituted by an operator and a vehicle tracking device monitoring trip parameters, the manufacturer of the GPS chip provides data in binary format.

The following commercially available and popular GPS receivers were tested—Table 1.

Because of the characteristics of Windows 10, its stability and the need for removing any Bluetooth interference, satellite receivers using a USB interface were selected. However, the software has any limitation concerning communication interfaces, and supports devices connected via USB, RS 232C, and Bluetooth. Several test sets were carried out in four positioning modes:

1. static (standalone) in an open area;
2. dynamic (real time kinematic—RTK) at minimum speed of 100 km h^−1^ on a dual carriage way;
3. RTK at maximum speed of 50 km h^−1^ in a built up area;
4. static in a built up area.

## 3. Results and Discussion

**Figure 1:**
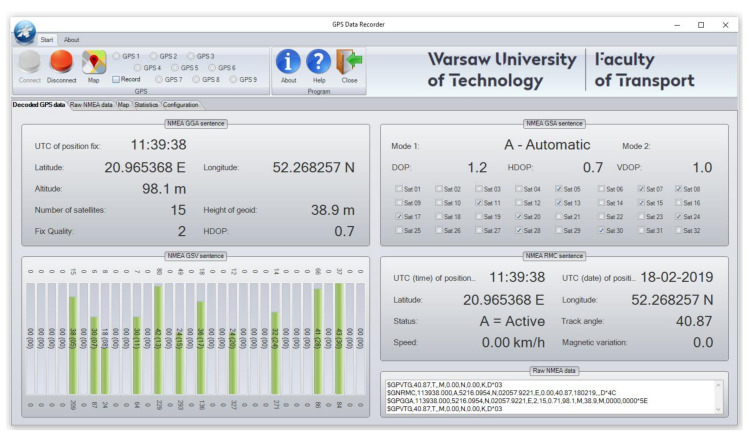
Home screen of GPS Recorder utility software.

The GPS data, received through NMEA protocol, are suitable for processing. Most commercially available navigation software uses a map-based user interface, which provides poor information on the positioning accuracy and GPS quality but shows the number of visible satellites and the strength of signal: (weak, good, or very good). Furthermore, the above software can sometimes be more rudimentary. A utility software offers much better capabilities to determine the positioning accuracy and GPS quality of the data sensed by GPS receivers. In fact, instead of displaying the detected positions on a map, it represents all data received from GPS in NMEA protocol. An example of such a software is Visual GPS (http://www.visualgps.net/#visualgpsview-content), which allows the user to receive data in GPS format, so that they can be saved in the hard drive, decoded, and graphically represented. Unfortunately, utility software also has its limitations, such as the ability to connect only to one GPS receiver. This considerably hampers data acquisition from devices used, and decreases the reliability of the performed analysis, in terms of asynchronous recording of received data. Therefore, an original utility software called GPS Recorder (a literal translation from Polish language) (Figure 1) was developed. It is compatible with Windows 10 and is capable of collecting position data from up to nine GPS receivers.

**Figure 2:**
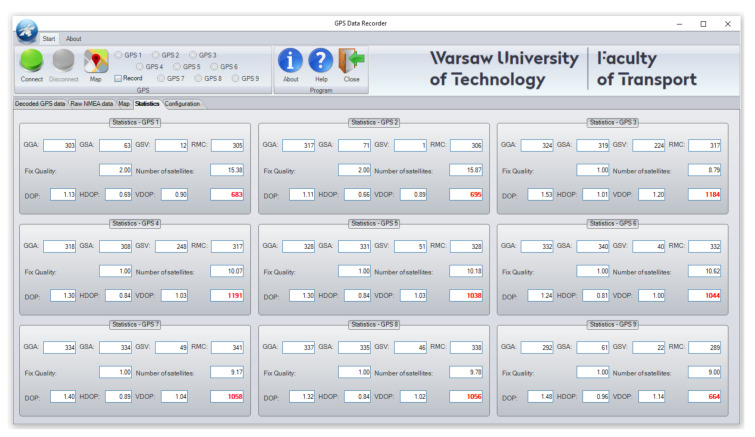
Statistical data screen of GPS Recorder utility software.

The software allows users to connect up to nine GPS receivers, acquire data in NMEA format, save them in the hard drive, decode and plot them, based on GGA, GSA, GSV, and RMC output message sequences and basic statistical calculations, mainly concerning GPS quality and positioning accuracy (Figure 2).

### 3.1. Accuracy Analysis of GPS Receivers

The following commercially available and popular GPS receivers were tested: Skytraq V6, Syngio BU353 S4, Hama SiRF STAR III, Holux M-215+, and Holux GR-213. They are hereafter referred to as A, B, C, D, E, F, G, H and I in no particular order. Because of characteristics of Windows 10, its stability and the need to eliminate any Bluetooth interference, receivers using a USB interface were selected. Please note that the software itself does not have any limitations concerning communication interfaces and supports devices connecting via USB, RS232C, and Bluetooth.

Several test sets were conducted for four cases:

1. Stationary in an open area,
2. At minimum speed of 100 km/h on a dual carriageway,
3. At maximum speed of 50 km/h in a built-up area,
4. Stationary in a built-up area.

**Figure 3:**
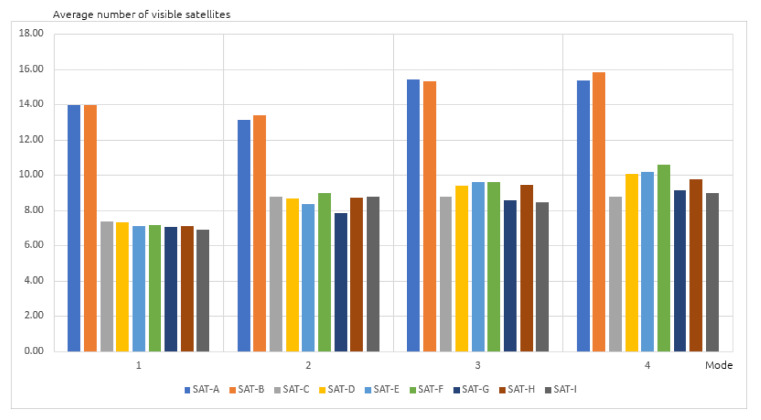
Average number of visible satellites in four positioning modes.

The number of satellites visible to the GPS receivers was first determined in each of the four positioning modes above. The recorded data (Figure 3) showed that the A and B receivers were superior: most satellites were visible to both of them, up to twice as many in the static positioning mode in an open area, while in the other modes they were able to see about 50% more than the other receivers. In fact, the A and B receivers are latest generation devices, using dual frequency (L1 and L2, i.e., 122,760 MHz). The higher average number of received GPS signals in the positioning modes 2, 3, and 4, compared to scenario 1, can be explained by the partial obstruction of the satellite signals by the vehicle. Substantial differences exceeding 10% (the number of satellites) in the results were observed between not only different receivers, but also devices of the same type.

**Figure 4:**
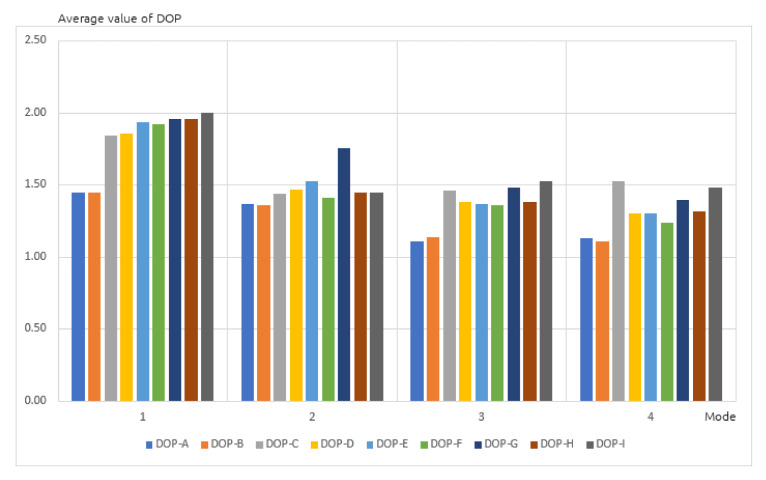
Average value of dilution of precision (DOP) in four positioning modes.

**Figure 5:**
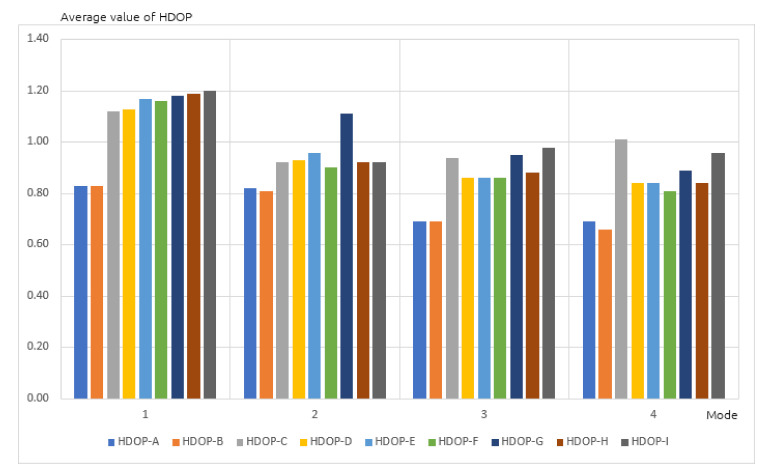
Average value of horizontal DOP (HDOP) in four positioning modes.

**Figure 6:**
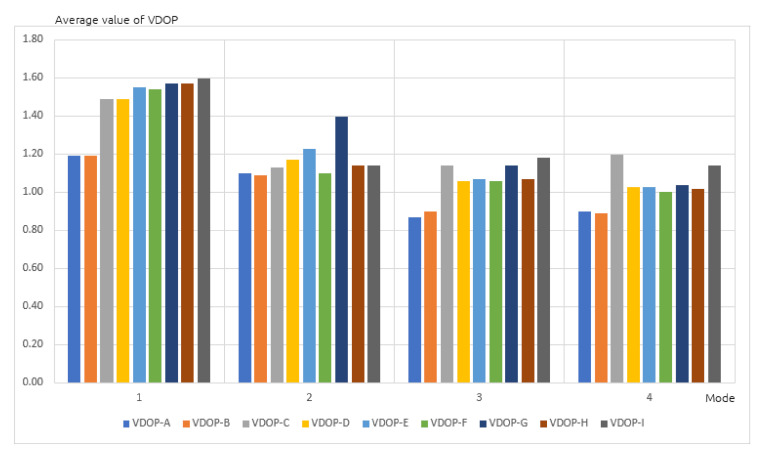
Average value of VDOP in four positioning modes.

During the next stage, average DOP values were analyzed and plotted on a chart (Figure 4). All receivers recorded values lower than 2 threshold, which guarantees a correct positioning. As previously, the best (lowest) values were achieved by the A and B receivers, used in static positioning mode in a built up area. Differences in the results were observed again, not only between different receivers, but also between devices of the same type. For the G and H receivers these differences were higher and exceeded 20% of DOP. Similar results were observed on the charts representing average values of HDOP (Figure 5) and VDOP (Vertical Dilution of Precision), related only to altitude (Figure 6) for the same four positioning modes. Once again, differences in the results occurred not only between different receivers, but also devices of the same type.

### 3.2. Reliability Analysis of GPS Receiver

**Figure 7:**
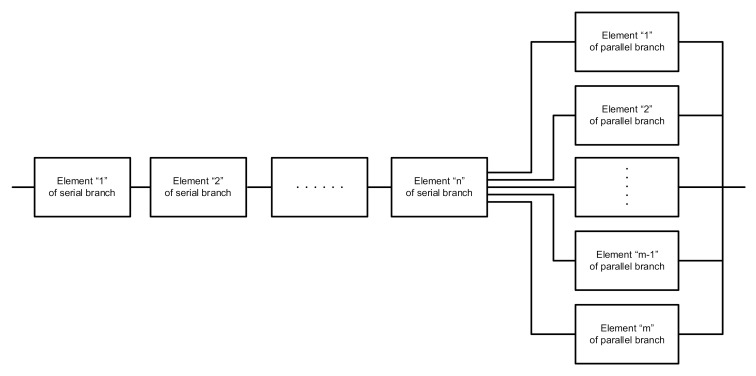
General reliability scheme of GPS receivers (Adapted from [39]).

After testing the above nine GPS receivers by means of GPS Recorder utility software, it could be deduced that the reliability structure is of mixed type, i.e., both serial and parallel, as outlined in Figure 7. Such an assumption was made for the needs of the analysis of the considered system consisting of the GPS Recorder software and GPS receivers. It is possible to adopt a different reliability structure if there is a redundancy of, for example, computers with GPS Recorder utility software installed.

**Figure 8:**
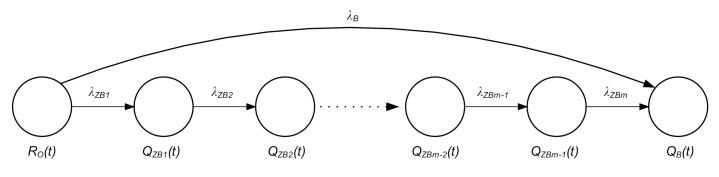
Within-system relationships from the viewpoint of safety (Adapted from [39]).

The failure of any element of the serial branch (e.g., failure of the computer running the GPS Receiver program) switches the system from the state of full operational capability *R_O_*(*t*) to that of failing security *Q_B_*(*t*). The failure of any element of the parallel branch (e.g., failure of the GPS receiver power supply, or no processing of the GPS signal by the receiver) switches the system from the state of full operational capability *R_O_*(*t*) to that of security threat *Q_ZB_*(*t*). Figure 8 describes the within-system relationships from the viewpoint of safety.

Designations on Figure 8:

*R_O_*(*t*)—the probability function of the system in the state of full operational capability,
*Q_ZB_*(*t*)—the probability function of the system in the state of safety threat,
*Q_B_*(*t*)—the probability function of the system in the state of safety breach,
*λ_B_*—equivalent change rate of serial branch elements,
*λ_ZB_*—change rate of parallel branch elements.

The following formula was derived after analyzing the aggregate system shown in Figure 8.

(1) $$\lambda_{B}=\sum_{i=1}^{n}\lambda_{Bi}$$

Chapman Kolmogorov equations describing the system shown in Figure 8.

(2) $$\begin{array}{l} R_{0}^{\prime }(t)=-\lambda_{B}\cdot R_{0}^{}(t)-\lambda_{ZB1}\cdot R_{0}^{}(t)\\ Q_{ZB1}^{\prime }(t)=\lambda_{ZB1}\cdot R_{0}^{}(t)-\lambda_{ZB2}\cdot Q_{ZB1}^{}(t)\\ Q_{ZB2}^{\prime }(t)=\lambda_{ZB2}\cdot Q_{ZB1}^{}(t)-\lambda_{ZB3}\cdot Q_{ZB2}^{}(t)\\ \ldots \\ Q_{ZBm-1}^{\prime }(t)=\lambda_{ZBm-1}\cdot Q_{ZBm-2}^{}(t)-\lambda_{ZBm}\cdot Q_{ZBm-1}^{}(t)\\ Q_{B}^{\prime }(t)=\lambda_{B}\cdot R_{0}^{}(t)+\lambda_{ZBm}\cdot Q_{ZBm-1}^{}(t) \end{array}$$

Given initial conditions:

(3) $$\begin{array}{l} R_{0}^{}(0)=1\\ Q_{ZB1}^{}(0)=Q_{ZB2}^{}(0)=\ldots =Q_{ZBm-1}^{}(0)=Q_{B}^{}(0)=0 \end{array}$$

After applying Laplace transformation the following system of equations was obtained:

(4) $$\begin{array}{l} s\cdot R_{0}^{*}(s)-1=-\lambda_{B}\cdot R_{0}^{*}(s)-\lambda_{ZB1}\cdot R_{0}^{*}(s)\\ s\cdot Q_{ZB1}^{*}(s)=\lambda_{ZB1}\cdot R_{0}^{*}(s)-\lambda_{ZB2}\cdot Q_{ZB1}^{*}(s)\\ s\cdot Q_{ZB2}^{*}(s)=\lambda_{ZB2}\cdot Q_{ZB1}^{*}(s)-\lambda_{ZB3}\cdot Q_{ZB2}^{*}(s)\\ \ldots \\ s\cdot Q_{ZBm-1}^{*}(s)=\lambda_{ZBm-1}\cdot Q_{ZBm-2}^{*}(s)-\lambda_{ZBm}\cdot Q_{ZBm-1}^{*}(s)\\ s\cdot Q_{B}^{*}(s)=\lambda_{B}\cdot R_{0}^{*}(s)+\lambda_{ZBm}\cdot Q_{ZBm-1}^{*}(s) \end{array}$$

By using inverse transformation the following equation was obtained:

(5) $$R_{0}(t)=e^{-(\lambda_{B}+\lambda_{ZB1})\cdot t}$$

(6) $$Q_{ZB1}(t)=\lambda_{ZB1}\cdot \left[\frac{e^{-(\lambda_{B}+\lambda_{ZB1})\cdot t}-e^{-\lambda_{ZB2}\cdot t}}{\lambda_{ZB2}-\lambda_{B}-\lambda_{ZB1}}\right]$$

(7) $$Q_{ZB2}(t)=\lambda_{ZB1}\cdot \lambda_{ZB2}\cdot \left[\begin{array}{l} \frac{e^{-(\lambda_{B}+\lambda_{ZB1})\cdot t}}{\left(\lambda_{B}+\lambda_{ZB1}-\lambda_{ZB3}\right)\cdot \left(\lambda_{B}+\lambda_{ZB1}-\lambda_{ZB2}\right)}-\\ -\frac{e^{-\lambda_{ZB2}\cdot t}}{\left(\lambda_{B}+\lambda_{ZB1}-\lambda_{ZB2}\right)\cdot \left(\lambda_{ZB2}-\lambda_{ZB3}\right)}+\\ +\frac{e^{-\lambda_{ZB3}\cdot t}}{\left(\lambda_{ZB2}-\lambda_{ZB3}\right)\cdot \left(\lambda_{B}+\lambda_{ZB1}-\lambda_{ZB3}\right)} \end{array}\right]$$

(8) $$\begin{array}{l} Q_{ZBm-1}(t)=\lambda_{ZB1}\cdot \lambda_{ZB2}\cdot \ldots \cdot \lambda_{ZBm-1}\cdot {\left(-1\right)}^{m+1}\cdot \\ \cdot \left[\begin{array}{l} \frac{e^{-\left(\lambda_{B}+\lambda_{ZB1}\right)\cdot t}}{\left(\lambda_{B}+\lambda_{ZB1}-\lambda_{ZB2}\right)\cdot \left(\lambda_{B}+\lambda_{ZB1}-\lambda_{ZB3}\right)\cdot \ldots \cdot \left(\lambda_{B1}+\lambda_{ZB2}-\lambda_{ZBm}\right)}+\\ +\frac{e^{-\lambda_{ZB2}\cdot t}}{\left(\lambda_{ZB2}-\lambda_{B}-\lambda_{ZB1}\right)\cdot \left(\lambda_{ZB2}-\lambda_{ZB3}\right)\cdot \ldots \cdot \left(\lambda_{ZB2}-\lambda_{ZBm}\right)}+\ldots +\\ +\frac{e^{-\lambda_{ZBm}\cdot t}}{\left(\lambda_{ZBm}-\lambda_{B}-\lambda_{ZB1}\right)\cdot \left(\lambda_{ZBm}-\lambda_{ZB2}\right)\cdot \ldots \cdot \left(\lambda_{ZBm}-\lambda_{ZBm-1}\right)} \end{array}\right] \end{array}$$

(9) $$\begin{array}{l} Q_{B}(t)=\frac{\lambda_{B}}{\lambda_{B}+\lambda_{ZB1}}\cdot \left[1-e^{-\left(\lambda_{B}+\lambda_{ZB1}\right)\cdot t}\right]+\lambda_{ZB1}\cdot \lambda_{ZB2}\cdot \ldots \cdot \lambda_{ZBm-1}\cdot \lambda_{ZBm}\cdot \\ \cdot \left[\begin{array}{l} {\left(-1\right)}^{m}\cdot \left(\begin{array}{l} \frac{e^{-\left(\lambda_{B}+\lambda_{ZB1}\right)\cdot t}}{\left(\lambda_{B}+\lambda_{ZB1}\right)\cdot \left(\lambda_{B}+\lambda_{ZB1}-\lambda_{ZB2}\right)\cdot \left(\lambda_{B}+\lambda_{ZB1}-\lambda_{ZB3}\right)\cdot \ldots \cdot \cdot \left(\lambda_{B}+\lambda_{ZB1}-\lambda_{ZBm-1}\right)\left(\lambda_{B}+\lambda_{ZB1}-\lambda_{ZBm}\right)}+\\ +\frac{e^{-\lambda_{ZB2}\cdot t}}{\left(\lambda_{ZB2}-\lambda_{B}-\lambda_{ZB1}\right)\cdot \lambda_{ZB2}\cdot \left(\lambda_{ZB2}-\lambda_{ZB3}\right)\cdot \ldots \cdot \left(\lambda_{ZB2}-\lambda_{ZBm-1}\right)\left(\lambda_{ZB2}-\lambda_{ZBm}\right)}+\ldots +\\ +\frac{e^{-\lambda_{ZBm-1}\cdot t}}{\left(\lambda_{ZBm-1}-\lambda_{B}-\lambda_{ZB1}\right)\cdot \left(\lambda_{ZBm-1}-\lambda_{ZB2}\right)\cdot \left(\lambda_{ZBm-1}-\lambda_{ZB3}\right)\cdot \ldots \cdot \lambda_{ZBm-1}\cdot \left(\lambda_{ZBm-1}-\lambda_{ZBm}\right)}+\\ +\frac{e^{-\lambda_{ZBm}\cdot t}}{\left(\lambda_{ZBm}-\lambda_{B}-\lambda_{ZB1}\right)\cdot \left(\lambda_{ZBm}-\lambda_{ZB2}\right)\cdot \left(\lambda_{ZBm}-\lambda_{ZB3}\right)\cdot \ldots \cdot \left(\lambda_{ZBm}-\lambda_{ZBm-1}\right)\cdot \lambda_{ZBm}} \end{array}\right)+\\ +\frac{1}{\left(\lambda_{B}+\lambda_{ZB1}\right)\cdot \lambda_{ZB2}\cdot \lambda_{ZB3}\cdot \ldots \cdot \lambda_{ZBm-1}\cdot \lambda_{ZBm}} \end{array}\right] \end{array}$$

The obtained relationships can be applied in order to determine the probability that any system consisting of m GPS receivers has to be in the state of full operational capability *R_O_*, security threat *Q_ZB_*, and security breach *Q_B_*.

Through computer-aided simulation and calculations it is possible to determine relatively quickly how the reliability factors of the tested GPS receivers influence the whole system.

It is possible to compute the probability of each state, i.e., full operational capability R_O_, safety threat *Q_ZB_*, and safety breach *Q_B_* for a system consisting of i = 9 GPS receivers. This procedure was presented in the following example.

Example 1

The following values describing the analyzed system were considered:

- research time—1 year (given in hours [h]):
  (10) t=8760[h]
- failure rate of the device using GPS Recorder utility software *λ_B_*:
  (11) λB=1.076605930037⋅10−5[1h]
- type I GPS receiver failure rate *λ_ZB_*_1_:
  (12) λZB1=1.076605930037⋅10−5[1h]
- type II GPS receiver failure rate *λ_ZB_*_2_:
  (13) λZB2=9.51844850902409⋅10−6[1h]
- type III GPS receiver failure rate *λ_ZB_*_3_:
  (14) λZB3=8.2843256660771⋅10−6[1h]
- type IV GPS receiver failure rate *λ_ZB_*_4_:
  (15) λZB4=7.06340225092323⋅10−6[1h]
- type V GPS receiver failure rate *λ_ZB_*_5_:
  (16) λZB5=5.85539890268842⋅10−6[1h]
- type VI GPS receiver failure rate *λ_ZB_*_6_:
  (17) λZB6=4.66004503655881⋅10−6[1h]
- type VII GPS receiver failure rate *λ_ZB_*_7_:
  (18) λZB7=3.47707847998956⋅10−6[1h]
- type VIII GPS receiver failure rate *λ_ZB_*_8_:
  (19) λZB8=2.30624512757072⋅10−6[1h]
- type IX GPS receiver failure rate *λ_ZB_*_9_:
  (20) λZB9=1.14729861341341⋅10−6[1h]

According to dependencies (5 ÷ 9) for research time t = 8760 [h] the probability of the system in the particular state is given by:

- in the state of full operational capability *R_O_*(*t*):
  (21) $$R_{0}(t)=0.9009$$
- in the state of safety threat *Q_ZB_*_1_(*t*):
  (22) $$Q_{ZB1}(t)=0.0858620089045631$$
- in the state of safety threat *Q_ZB_*_2_(*t*):
  (23) $$Q_{ZB2}(t)=0.00360517529559324$$
- in the state of safety threat *Q_ZB_*_3_(*t*):
  (24) $$Q_{ZB3}(t)=8.77547595242873\cdot 10^{-5}$$
- in the state of safety threat *Q_ZB_*_4_(*t*):
  (25) $$Q_{ZB4}(t)=1.36543510888452\cdot 10^{-6}$$
- in the state of safety threat *Q_ZB_*_5_(*t*):
  (26) $$Q_{ZB5}(t)=1.40868180519132\cdot 10^{-8}$$
- in the state of safety threat *Q_ZB_*_6_(*t*):
  (27) $$Q_{ZB6}(t)=9.64790335381539\cdot 10^{-11}$$
- in the state of safety threat *Q_ZB_*_7_(*t*):
  (28) $$Q_{ZB7}(t)=4.19120993840803\cdot 10^{-13}$$
- in the state of safety threat *Q_ZB_*_8_(*t*):
  (29) $$Q_{ZB8}(t)=5.68497094181757\cdot 10^{-14}$$
- in the state of safety threat *Q_Z_*(*t*):
  (30) $$Q_{B}(t)=0.00954368142145555$$
- The reliability of the whole system is given by:
  (31) $$R_{S}(t)=R_{0}(t)+\sum_{1}^{8}Q_{ZBi}(t)=0.990456319$$

The obtained *R_S_* value is significantly higher than *R*_0_ and is valid for purposes of whole system analysis. The derived relationships were verified through completed calculations and validated in order to analyze and compare different types of integrated GNSS.

## 4. Conclusions

Popular and commercially available GPS receivers were used for this research. Although they are widely considered as accurate and reliable devices, there is little to substantiate this opinion, besides the common wisdom and brand reputation. There is no mechanism and, above all, no available software that allows users to objectively compare GPS receivers by the quality, stability, and reliability of information position data they provide. Now, such comparison is possible, by using GPS Recorder utility software that was developed for the purposes of this research. The acquired data proved correct the assumption that the best quality parameters were offered by the A and B receivers using dual frequency (L1/L2) satellite signals. They acquired the most satellites and offered the lowest DOP, HDOP, and VDOP values. However, under real operating conditions, the most similar to those of GPS receivers (i.e., driving in an open area and in a built-up area), the observed differences were much lower. In the case of driving at 100 km h^−1^ and more, they were practically negligible. In the case of single frequency (L1) receivers, the obtained results were comparable but also different enough to identify the E and F receivers as superior. At the same time, it can be concluded that differences were found both between different types of GPS receivers and devices of the same type.

Based on the reliability tests of nine GPS receivers, carried out by means of the above original utility software, it is advisable to use lower class devices in parallel configuration and analyze the NMEA data in the software itself.

These conclusions not only substantiate the validity of the undertaken research, but also drive their next directions. The developed software solution could well be used for further research on a wider group of same types of GPS receivers. The results of this study could lead to the draft of a procedure for evaluating and selecting GPS receivers, based on their quality, prior to use. This could have a paramount importance for uses in special purpose vehicles or transport telematics systems.

## Figures and Tables

**Table 1:** GPS Receivers Specification.

Parameters	Skytraq V6	Syngio BU353-S4	Hama	Holux M-215+	Holux GR-213
Chipset	Venus V6	SiRF StarIV	SiRF StarIII	MTK MT3333 GPS/GLONASS chipset	SiRF StarIII
Receiver Type	L1, C/A code 51-channel acquisition 14-channel tracking	L1, 1575.42 MHZ 48 all-in-view tracking	L1, up to 20 satellites	L1, 1575.42 MHz 66 parallel searching, 22 tracking channels	L1, up to 20 satellites
C/A Code	N/A	1.023 MHz	N/A	1.023 MHz	N/A
Maximum Update Rate	10 Hz	1 Hz	1 Hz	1 Hz	1 Hz
Accuracy	Position 2.5 m CEP Velocity 0.1 m/s Time 250 ns	< 2.5 m 2D RMS SBAS Enabled	up to 2 m (WAAS)	Non DGPS (Differential GPS): Position: 3.0 m CEP excluding SA Velocity: 0.1m/s. Interval: 0.1 µs to Sync GPS DGPS (EGNOS/WAAS/ MSAS): 2.5 M	Non DGPS (Differential GPS) Position 5–25 m CEP without SA Velocity 0.1 m/s, without SA Time 1 μs sync GPSTime EGNOS/WAAS: Position < 2.2 m, horizontal 95% of time < 5 m, vertical 95% of time
Time To First Fix	Hot-Start < 1 s Warm-Start 25 s average Cold-Start 29 s average	Hot-Start 1 s average Warm-Start 35 s average Cold-Start 35 s average	Hot-Start < 1 s Warm-Start 35 s Cold-Start 42 s	Hot-Start 1 s Warm-Start 30 s Cold-Start 31 s	Hot-Start 1 s Warm-Start 38 s Cold-Start 42 s
Sensitivity	−161 dBm	−163 dBm	−159 dBm	−165 dBm	−159 dBm
Protocol	NMEA-0183 v3.01 Secondary: SkyTraq Binary	NMEA0183 Secondary: SiRFbinary	NMEA-0183 v.3.00	NMEA-0183 v3.01	NMEA-0183 v2.2
Interface	USB 2.0	USB 2.0	USB 2.0	USB 2.0	USB 2.0
Supply Voltage	1.2 V, 3.3 V	5.5 V (USB)	5.5 V (USB)	5.5 V (USB)	5.5 V (USB)
Current Consumption	Acquisition ~50 mA Tracking ~23 mA	55 mA Maximum	N/A	N/A	N/A
Operating Temperature	−40 °C~85 °C	−40 °C~85 °C	−40 °C~85 °C	−10 °C~65 °C	−10 °C~65 °C
Dimension	38.0 × 40.5 × 12.3 mm	53.0 × 19.2 mm	45.0 × 37.0 × 20.0 mm	64.5 × 42 × 17.8 mm	64.5 × 42 × 17.8 mm
Weight	N/A	62.37 g	70.0 g	84.0 g	84.0 g
